# Rising of a global silent killer: critical analysis of chronic kidney disease of uncertain aetiology (CKDu) worldwide and mitigation steps

**DOI:** 10.1007/s10653-022-01373-y

**Published:** 2022-09-12

**Authors:** Watte Vidanelage Dinesha Priyadarshani, Angela F. Danil de Namor, S. Ravi P. Silva

**Affiliations:** 1 Advanced Technology Institute, University of Surrey, Guildford, UK; 2 Department of Chemistry, University of Surrey, Guildford, UK

**Keywords:** CKDu, Health impact, Prevalence, Causes and mitigation, Nephrotoxins, Polluted water and soil

## Abstract

Chronic kidney disease of uncertain aetiology (CKDu) is an advanced version of chronic kidney disease (CKD) which bears a high burden on the world health economy. More than 200 articles were analysed to understand the disease responsible for more than 30,000 deaths per year. CKDu is a non-communicable occupational disease that has a progressive deterioration of the kidney in the absence of CKD risk factors such as hypertension, diabetes and glomerulonephritis, while the diagnosis is only possible at the later stages when kidney function is no longer effective. Published evidence for the existence of CKDu was found for around 35 countries. This is a growing health issue in Asia, Central America, Africa and Middle East with identified hot spots. Despite many research studies over decades, the exact root causes are still uncertain. Six main suspected causative factors are identified. Those are heat stress, strenuous labour, dehydration, use of agrochemicals, exposure to heavy metals and the use of polluted water and agricultural lands. This review summarizes four key areas which are CKDu and its general medical background, worldwide prevalence, suspected causative factors and potential circumventing steps to mitigate against CKDu. The importance of further studies addressing early detection and surveillance methods, contribution of nephrotoxins in environmental health, soil chemistry on transporting nephrotoxins, geological parameters which influence the prevalence of the disease and other related sectors to overcome the mysterious nature is highlighted. Mitigation steps to lessen the burden of CKDu are also identified.

## Introduction

Chronic kidney disease of uncertain aetiology (CKDu) is an insidious occupational endemic problem involving the progressive deterioration of kidney function leading to ultimate fatalities with huge impact on the world health economy. CKDu cases are reported all around the world, and a full understanding of the situation is still not in place. A number of names can be found based on several factors such as observed region: Mesoamerican nephropathy (MeN), chronic kidney disease of unknown aetiology (CKDu), chronic kidney disease of non-traditional origin/cause (CKDnt), Mesoamerican endemic nephropathy (MEN), Balkan endemic nephropathy (BEN), agricultural nephropathy, unspecified chronic kidney disease (unCKD), non-diabetic end-stage renal disease (ndESRD), Chronic Agricultural Nephropathy (CAN), Chronic Kidney Disease of multifactorial origin (CKD-mfo), chronic interstitial nephritis in agricultural communities (CINAC) and Sri Lankan agricultural nephropathy (SAN) (Jayasumana et al. 2015a, b, c, d;^ 2016^; Orantes et al. 2014; Rajapakse et al. 2016; Wijkstrom et al. 2017; S. J. Wimalawansa, 2016). Even though CKDu is much similar to CKD which too is a heavy burden to the world health economy, the CKDu diagnosis can only be performed at the end-stage renal disease (ESRD) when no steps can be taken to treating the damaged kidney. Diagnosing for CKDu is a death warrant given that available treatments are not affordable by affected communities.

**Fig. 1:**
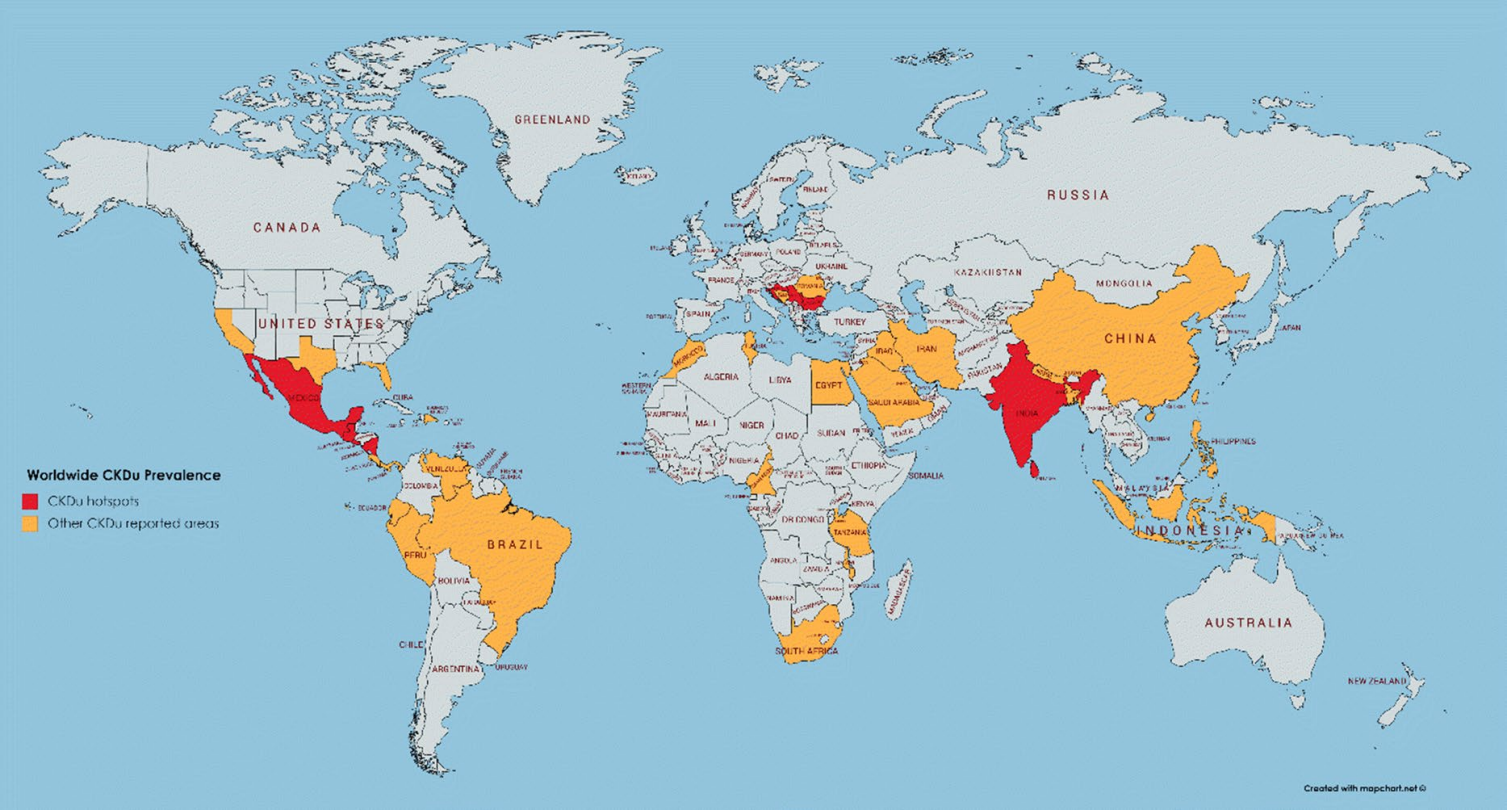
Worldwide CKDu prevalence. Red colour for CKDu hot spots and orange colour for other CKDu reported countries

Initially, CKDu was limited to tropical region countries, but according to recent reports, this disease is spreading rapidly beyond that. CKDu was first discovered in Balkan countries in the 1960s and then in Central America but it was first identified in Sri Lanka in the 1990s (Cheryl Dybas, 2018; Gifford et al. 2017; S. A. Wimalawansa & Wimalawansa, 2016). Since then, the number of people affected by CKDu has increased notably to an extent that in the last three decades it has expanded to 35 countries causing in excess of 40,000 deaths in Mesoamerica and Sri Lanka alone. This number of victims is higher than the death toll produced by famous Ebola virus (Jayasumana et al. 2014) (La Isla Network, 2020). Recently, it has been estimated that the world CKDu death toll is more than 30,000 per year (S. J. Wimalawansa & Dissanayake, 2019). Countries facing this problem are shown in Fig. 1. The irony of CKDu at present is that although the world possesses advanced technologies, due to the unavailability of early diagnosis, it is not possible to combat the disease which progresses steadily remaining as a silent killer. Furthermore, this disease targets humans in the productive age group. So far, the disease has emerged mainly among poor male agricultural workers in developing countries but developed countries are also not immune because of the global trade mechanism and some causative factors can be transferred via trades such as food. However, without concerted effort, the disease will engulf the whole world with the upcoming water stress and rapid climate change. Hence, the sooner the disease is eradicated, the better.

From the beginning, scientists all around the world are trying to understand the behaviour of the disease together with its risk factors and causative factors to provide a promising solution. Unfortunately, no exact causative factors, risk factors or early diagnosing methods have presented itself so far leaving the origin of the disease a mystery which needs to be unfold. It is suspected that the lack of attention towards the importance of research studies and funding for the CKDu issue may be a major reason for this failure. However, according to the reported studies, the disease has many roots needed to be eradicated which require combined expertise roles in multiple sectors such as nephrology, environmental health, geochemistry, chemical engineering, politics and industrial performance. This review intends to address many of the facts/studies which have been carried out so far under various organisations such as medical and environmental parameters. We believe that the lack of cohesive understanding or knowledge distribution among scientists, policymakers and industrial roles acts as a barrier towards a promising cure and efforts in addressing all the scientific facts in simple terminology towards its mitigation. Therefore, this paper is a result of general analysis of the CKDu situation addressing medical definitions and health effects, its worldwide prevalence, suspected causative factors, and mitigating steps.

## Methodology

Articles related to CKDu were searched through a number of databases such as PubMed and ScienceDirect. The search was done under different topics related to CKDu including synonyms of the disease to collect data about medical facts, prevalence, environmental effects, and anthropogenic involvement to collect most of the data to understand the big picture of CKDu. The search was done with no date or region/country limits. Collected articles were critically analysed and overall and potential facts were highlighted towards mitigation of the disease. More than 200 articles which are mostly related to CKDu were filtered out from the collected reporting case studies and reviews. Information was presented under four key categories namely medical definition and health effects of CKDu, worldwide prevalence of CKDu, suspected causative factors and mitigation steps for CKDu. Most relevant articles in this manuscript were selected for further understanding of the key areas of CKDu mitigation.

## Results

### Medical definition and health effects of CKDu

Kidneys are a pair of bean-shaped organs in the abdominal cavity centring the spinal cord playing a vital role in the human body. Its main function is to clean the blood and form urine. By doing this, the kidney will maintain homeostasis, waste excretion, pH balance (preventing acidemia/alkalemia), osmolality and blood pressure. Nephrons are the basic functional and structural renal units located in pyramid shaped lobes inside the kidney and there are about a million nephrons in one kidney. Working mechanism of the nephron involves two steps which are ultrafiltration at glomerulus and reabsorption at tubule. Afferent arteriole brings uncleansed blood to glomerulus (a cluster of blood vessels) where ultrafiltration is performed via a pressure driven mechanism. At this stage, almost all of the components in blood except proteins and blood cells are gone through the skins of glomerulus and Bowman’s capsule and comes into the tubule. Then, required nutrients, minerals and water are reabsorbed by blood vessels, while wastes (mainly Urea and Uric acid) and excess components move forward to the collecting duct creating urine (Rayner et al. 2016; Waugh & Grant, 2001). Due to medical and environmental factors, this working mechanism can be interrupted in many ways generating kidney diseases with immplications on lifestyles.

A number of kidney diseases can be found such as chronic kidney disease (CKD), glomerulonephritis, polycystic kidney disease and kidney stone. Among these, CKD gets more attention due to its common nature and vast burden to the world health economy. It is defined with loss of kidney function progressively over a long period of time (Chronic Kidney Disease—World Kidney Day, 2022). The kidney function can be lost up to 90% without having any symptoms. CKD has well-known risk factors such as diabetes, hypertension, snake bites, glomerulonephritis and urological diseases (Rajapakse et al. 2016). If proper surveillance is in place, early detection is mostly possible and treatments can be done to halt further deterioration (Chronic Kidney Disease—World Kidney Day, 2022). But advanced or end-stage CKD leaves more probability towards death due to lack of treatments. Furthermore, the kidney has reduced or no function at that time and patients face an extremely painful situation. Chronic kidney disease of uncertain aetiology (CKDu) is quite similar to the end stage of CKD. CKDu is defined based on the absence of known risk factors of the normal chronic kidney disease (CKD) such as diabetes and hypertension, while tubular proteinuria, absence of hypertension and oedema, characterize the disease clinically (Noble et al. 2014; Cooray et al. 2019). Microscopic analysis of renal tissues (histology of renal biopsy) of CKDu patients has showed tubulointerstitial disease (Athuraliya et al. 2011). A kidney biopsy study of sugarcane workers in Nicaragua has confirmed that CKDu comes with glomerular and tubulointerstitial damage along with glomerulosclerosis and chronic glomerular ischaemia (Wijkstrom et al. 2017). Some symptoms can be fatigue, panting, lack of appetite, nausea, sleep disturbance, exhaustion, weight loss and death. Unfortunately, it is not possible to identify the disease in the early stages, making the diagnosis a death warrant because kidneys are non-functional by then due to severe irreversible damage (Cooray et al. 2019; La Isla Network, 2020; Noble et al. 2014; Redmon et al. 2014). Initially, it was observed among male farmers age 40–60 predominantly but now younger people are affected by the disease making the situation more dangerous (Cheryl Dybas, 2018; Noble et al. 2014; Rajapakse et al. 2016).

There are two biological tests similar to those used to detect CKD which are blood and urine analyses. **Blood analysis**: Creatinine is a waste product of muscle functions. It is transported via blood, filtered out and excreted in the kidney. Blood samples are tested for creatinine level and higher value means lower kidney function. Furthermore, the level of creatinine in blood is used to calculate the GFR (glomerular filtration rate) which measures the kidney function generating information about the presence and extent of abnormalities. Normally, a healthy kidney has a GFR value of 90 mL/min or above. In addition the sugar level of the blood is also tested (Chronic Kidney Disease—World Kidney Day, 2022). **Urine analysis**: Creatinine level and presence of proteins such as albumin are considered. A healthy kidney does not filter out proteins from the blood and the presence of proteins in the urine indicate the deterioration of kidney function. This is a simple dipstick test (Chronic Kidney Disease—World Kidney Day, 2022). CKDu has four stages based on proteinuria and estimated GFR (mL/min/1.73 m^2^) which are > 90, 60–89, 30–59 and < 30 (Rajapakse et al. 2016). However, many reports indicate that in CKDu, proteinuria (protein in urine) is not observed significantly until the final stages of the disease (La Isla Network, 2020; Redmon et al. 2014). So studies should be carried out to find specific screening and detection methods. Currently, someone gets suspected as a CKDu patient if blood test shows abnormality in kidney function without having diabetes or high blood pressure.

Unfortunately, when CKDu is diagnosed, irreversible damage has already happened to the kidney and the kidney is not able to clean the blood to an effective level, concentrating waste materials in the blood that can damage other organs. In this case, cleaning the blood should be done artificially (dialysis) or a suitable kidney from another human should be exchanged with the deteriorated kidney (kidney transplantation) in order to protect the body function and these treatments are called renal replacement treatments (RRT) (Chronic Kidney Disease–World Kidney Day, 2022). **Dialysis**: Two types are available naming haemodialysis and peritoneal dialysis. In haemodialysis, blood will circulate through a machine which cleans the blood. This can be done in either at dialysis centre (3–5 h per session and three sessions per week) or at home (3–10 h per session and 3–7 sessions per week). In peritoneal dialysis, blood is not circulating out the body but addition of a clean fluid to the abdominal cavity that adsorbs the waste products from the blood through vessels is done. Later, this fluid is drained out from the body. Often this treatment is done at home (Chronic Kidney Disease-World Kidney Day, 2022). **Kidney transplantation:** This treatment involves transplanting a healthy kidney from a person to another who has a deteriorated kidney. This will give a proper solution, but availability of a suitable healthy kidney is very rare, and it needs specific requirements such as compatible blood group. This treatment is expensive, and a long waiting time is often observed. Furthermore, until all of the requirements are fulfilled, regular dialysis needs to be carried out (Chronic Kidney Disease - World Kidney Day, 2022). Summary of the medical facts is shown Table 1.

**Table 1:** Definition, symptoms, diagnosing techniques and treatments

CKDu definition	Loss of kidney function progressively over a long period of time where absence of known risk factors of the normal chronic kidney disease (CKD) such as diabetes and hypertension while tubular proteinuria, absence of hypertension and oedema, characterize the disease clinically
Symptoms	Fatigue, panting, lack of appetite, nausea, sleep disturbance, exhaustion, weight loss and death
Diagnosing techniques	Blood analysis (creatinine) and urine analysis (creatinine level and presence of proteins)
Treatments	RRT: Dialysis (Haemodialysis and Peritoneal Dialysis) and Kidney transplantation

Psychological health plays a very important role as well. Most of the patients are economically poor agricultural workers and the cost of RRT is not affordable for the reported CKDu folk communities. Lack of physical strength due to the disease, people are unable to do farming or day today works being a burden to the family. The feeling of being a burden at their productive age, spending saved money for treatments and not having a hope of permanent cure cause a huge mental stress and some even choose the suicide over the life battle. Some anecdotal researches indicate that marriages as well are become difficult to these communities because other communities are refusing to give their children in fear of CKDu. Hence, CKDu poses a threatening and painful situation in both physical and psychological viewpoints. The best way to tackle the situation is to eradicate the causal roots of the disease, since early detection is difficult so far.

### Worldwide prevalence of CKDu

As far as research efforts are concerned, South Asia and Mesoamerica are the regions which so far have made the major contributions to tackle the disease. There are 35 countries found as effected by CKDu and those are divided into seven regions for the clarity of present. Those are Central America, Asia, North America, South America, Balkan Countries, Africa and Middle East.

Central America lies between 0 and 30 N latitudes land-bridging the North and South American continents. The coastal region (west by the Pacific Ocean and east by the Caribbean Sea) and the much cooler inland mountain chain can be observed. Human population is mostly found in the mountain region. The economy is based on agriculture mainly on tropical fruits, rice, coffee, corn and sugarcane and a tropical climate is observed. (World Regional Geography, n.d., 2022) CKDu affected countries in Central America are El Salvador, Costa Rica, Guatemala, Nicaragua and Panama, while the most exposed communities are sugarcane workers, miners, construction workers and farmers. People in the coastal region are more affected than those in the mountainous region (Gifford et al. 2017) (Map of CKDu Worldwide Prevalence – Just Another Your LaIsla Multisite Install Site, 2020). Young and middle-aged workers in sugarcane industry are mainly affected by the CKDu. Sugarcane cutting involves tremendous cardiovascular demand under very hot climatic conditions over 6 months of harvest period (November–April) (Bodin et al. 2016). La Isla is a region in Nicaragua having the meaning of Island of widows by its name. In this region, most of the men have died due to CKDu leaving more widows (Map of CKDu Worldwide Prevalence—Just Another Your LaIsla Multisite Install Site, 2020). There are lots of well-written documents which provide evidence for the observation of CKDu or MeN throughout the Central America. Reported death toll of CKDu in Central America is in the order of tens of thousands though all the deaths are not counted properly (Wesseling et al. 2020). A study carried out on a rural coastal region called Bajo Lempa reported two third of studied cases of CKDu over a 10 year period (2004–2013) where the major effect is observed on males in their productive age over females (Garcia-Trabanino et al. 2016). In 2011, in El Salvador, CKD was the first cause of men deaths and the fifth in women while a prevalence study of 2388 persons showed the predomination of CKDu among those CKDs (Orantes et al. 2014). In Costa Rica, Guanacaste is the hottest region  in which an excess mortality has been observed over a period of more than four decades due to CKDu and CKD (1970–2012) (Wesseling et al. 2015).

Countries affected by CKDu in Asia are Sri Lanka, India, Bangladesh, Nepal, Taiwan, China (Xing et al. 2015), Indonesia and Philippines. The highest number of people affected by this disease are in Sri Lanka and India, while others seem to be less advanced in scientific studies, but all have been able to detect the presence of the disease. In Sri Lanka, it was initially observed that male farmers in the age range 30–40 were predominantly affected by the disease, while at present, the trend has moved towards the younger population making the situation more dangerous (Noble et al. 2014; Rajapakse et al. 2016). Rice farmers are the ones mainly affected. CKDu was first observed in the 1990s, and over these three decades, its rapid prevalence in North Central province, Uva province and North Western province in the dry zone can be clearly noticed in Sri Lanka. In 2014, 16,479 CKDu cases were reported, and by 2015, it was increased to 20,828 evidencing its rapid prevalence (Cheryl Dybas, 2018). A study shows that 70.2% of CKD patients were diagnosed with CKDu, while the rest were due to known CKD risk factors such as diabetes and hypertension (Jayasekara et al. 2015). There are a number of joint mitigation studies carried out by the WHO and the National Science Foundation (NSF) in Sri Lanka (WHO, 2016). In India, poor agricultural communities are mostly affected. A study reports 16% of CKDu observations in 2012 while stating the patients of CKDu are younger than those from diabetic nephropathy (Map of CKDu Worldwide Prevalence – LaIsla Multisite Install Site, 2020; Rajapurkar et al. 2012).

Reported CKDu countries in North America are USA (California, Texas, Florida), Mexico and Dominican Republic. A study carried out in 2016 indicates that agricultural communities are affected with the presence of CKDu in California along with AKI (Acute Kidney Injuries). However, this report does not provide a clear evidence of the facts leading to the disease (Moyce et al. 2016). As far as South America is concerned, CKDu cases are found in Brazil, Ecuador and Peru. A study involving 90,356 patients from National Renal Replacement Therapies Database in 2000–2004 showed that the main cause for CKD is unknown aetiologies, while others are diabetes, hypertension and glomerulonephritis (Cherchiglia et al. 2010). CKDu also affects Balkan countries such as Serbia, Bulgaria, Romania, Croatia and Bosnia (Elledge et al. 2014). Many reports indicate that some of these countries are CKDu hot spots, but lack of research studies do not allow to have a clear picture of CKDu. Some reports Balkan endemic nephorapathy (BEN) is type of a CKDu which has been solved the root of the problem with the involvement of the plant *Aristolochia sp.* (Gifford et al. 2017).

There are number of countries affected by CKDu in Africa (South Africa, Tanzania, Cameroon, Morocco, Tunisia and Malawi). A study in 2014 reports that half of the CKD cases (49.1%) were of unknown aetiologies evidencing its presence in Northern Tanzania, while another study in a referral hospital in Cameroon reports 14.7% unknown aetiology ESRD (end-stage renal disease) cases (Halle et al. 2015; Stanifer et al. 2015). CKDu is also a matter of concern in Middle East Countries (Saudi Arabia, Iran, Qatar and Egypt). In Egypt, a study was carried out. In doing so, 1004 regular haemodialysis patients from the 2136 cohort in El-Sharkia Governorate, Egypt were randomly selected with the aim of assessing the epidemiology and risk factors for end-stage renal disease (ESRD) showing that diabetes and hypertension are the main risk factors, while kidney stones, urinary tract infection, congenital abnormality and glomeronephritis also play a significant role in the disease. Curiously, a higher fraction (17.7%) showed the involvement of uncertain aetiologies evidencing the presence of unknown aetiology of ESRD or CKDu. Furthermore, it was reported that the values of unknown aetiology of ESRD for El-Minya governorate—27%, Cairo governorate—18.1% and Menoufiya governorate—20.6%. In addition Iran—14.4%, Qatar—14% and Saudi Arabia—19.9% had shown the CKDu prevalence (Ghonemy et al. 2016). Another study reports a survey of ESRD prevalence and aetiologies in the El-Minia Governorate, Upper Egypt in 2006 among patients who use RRT. A number of aetiologies have been found such as hypertension, chronic glomerulonephritis, diabetic nephropathy, obstructive uropathy, bilhaziasis, analgesic nephropathy, chronic pyelonephritis, while highest contribution shows no aetiologies. This also records the presence of unknown aetiology of ESRD or CKDu. Further, it reports the prevalence of CKDu as Upper Egypt Governorates—15.2%, Lower Egypt Governorates—11.5%, Canal Governorates—37.2% and Border Governorates—17.6%, while the mean age of patients were 46 ± 13 and male/female ratio is 65%:35% (El-Minshawy, 2011). Table 2 shows the worldwide CKDu prevalence.

**Table 2:** Worldwide CKDu prevalence

Region	Reported countries	Effected communities	Suspected causal factors	References
Central America	El Salvador, Costa Rica, Guatemala, Nicaragua, Panama	Mostly Sugarcane workers, Miners, construction workers, and farmers	Heat stress, Strenuous labour and dehydration, Pesticides	Orantes et al. (2014), Bodin et al. (2016), Wesseling et al. (2020), Garcia-Trabanino et al. (2016), Wesseling et al. (2015)
		(Pacific coastal region is more affected than midland/mountainous)		
Asia	Sri Lanka, India, Bangladesh, Nepal, Taiwan, China, Indonesia, Philippines	Mainly rice farmers and Chena farmers	Heat stress, Strenuous labour and dehydration, Consumption of heavy metal accumulated foods,	Cheryl Dybas (2018), Xing et al. (2015), Jayasekara et al. (2015), Rajapurkar et al. (2012), Balasooriya et al. (2022)
		(Lowland is more effected than midland or mountainous)		
			Consumption of water with high hardness and fluoride content, Use of low-quality aluminium kitchen utensils, Agrochemicals, Genetic links and Nephrotoxic mycotoxins	
North America	USA-California, USA-Texas, Florida, Mexico, Dominican Republic	–	–	Moyce et al. (2016)
South America	Brazil, Ecuador, Peru	–	–	Cherchiglia et al. (2010)
Balkan countries	Serbia, Bulgaria, Romania, Croatia, Bosnia	–	–	Gifford et al. (2017), Elledge et al. (2014)
Africa	South Africa, Tanzania, Cameroon, Morocco, Tunisia, Malawi	–	–	Halle et al. (2015, Stanifer et al. (2015)
Middle East	Qatar, Egypt, Saudi Arabia	–	–	Ghonemy et al. (2016), El-Minshawy (2011)

–indicate lack of relevant studies

### Suspected causative factors

**Fig. 2:**
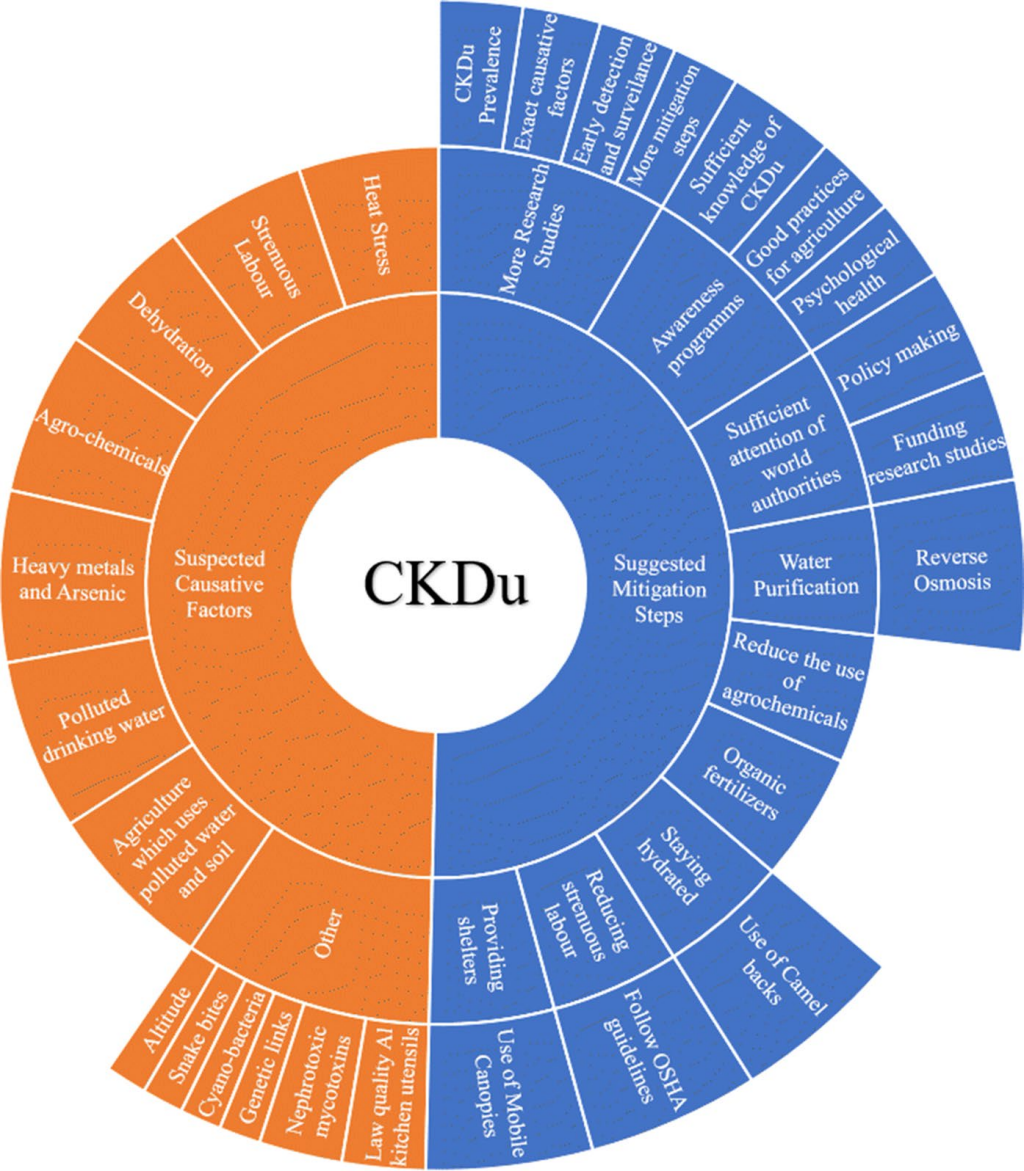
Suspected causative factors and suggested mitigation steps

As mentioned above, the exact causative factors are still unknown for the CKDu and it is suspected that it has multifactorial origin. There are several factors which can be grouped under four main headings. Figure 2 shows a summary of suspected causative factors.

### Heat stress, strenuous labour and dehydration

Heat stress occurs in the body due to overheating. Some symptoms can be profuse sweating, dizziness, heat rash, fainting and severe thirst. This can lead to dehydration unless a higher amount of water is consumed. Furthermore, strenuous labour also contributes towards sweating removing water via sweat rather than urine, decreasing the water level in blood and urine (Heat Stress | Environmental Health and Safety | Iowa State University, n.d., 2021) (Heat Stress—Temperature—HSE, n.d., 2021). An involvement of heat stress and dehydration in CKDu patients have been reported in El Salvador sugarcane cutters. The mean temperature before noon was 34–36 °C and after noon 39–42 °C. US OSHA (Occupational Safety and Health Administration) suggests a reduction of 70% in the working hours when the WBGT (Wet Bulb Globe Temperature) exceeds 30 °C which was not practically followed resulting in a high degree of heat stress in the body. In order to counteract this excessive heat and maintain the body temperature, sweating is the only option. Even though the mean liquid intake (0.8 L per hour) seemed to be enough to maintain serum osmolality, stress on renal reabsorption was higher. Osmolality, creatinine level and specific gravity of urine were increased, while pH was decreased indicating the dehydration. Furthermore, an increase in serum levels of creatinine, uric acid and urea nitrogen was observed (Garcia-Trabanino et al. 2015). An assessment of the heart rate of El Salvador sugarcane cutters showed that the job involves a very high cardiovascular demand and labours maintain this daily for a long period of time given that the harvest period is around 6 months (Lucas et al. 2015). In the Padaviya region in Sri Lanka where CKDu prevalence is higher, paddy farmers showed dehydration despite of farming or not and an acute dehydration upon working, according to the plasma and urine osmolality investigations. Paddy cultivation also requires strenuous labour under hot climatic conditions in this area (Nanayakkara et al. 2020).

### Agrochemicals: pesticides, herbicides and fertilizers

Agriculture is one of the main common activities in countries affected by CKDu with a possible exposure of workers to a variety of agrochemicals such as pesticides, herbicides and fertilizers. Main synthetic agrochemicals include glyphosate, 2,4-D and paraquet. A study shows that both CKDu and non-CKDu people in Girandurukotte and Medawachchiya in Sri Lanka has been subject to low level of neonicotinoid insecticides exposure such as imidacloprid and desmethyl-acetamiprid. These are neuro-active agents, and in the 2018, the EU banned imidacloprid use for outdoors (Kabata et al. 2016). Exposure to organochlorine pesticides was reported from India among both, CKDu and healthy patient controls which have been tested through blood analysis (Siddarth et al. 2014).

Glyphosate is an aminophosphonic acid/aminophosphonate which is widely used as herbicide while used as a ripener in sugarcane pre-harvest. First glyphosate appears as a descaling agent to remove mineral deposits from pipes and boilers due to its chelating and wetting abilities. It is readily soluble in water, while its half-life in normal water is limited to weeks but in hard water that abundantly found in CKDu areas, it can last from 7 to 22 years. Addition to consumption of contaminated water and foods, glyphosate can be dissolved in sweat and dermally adsorbed during the glyphosate preparations for field applications (Jayasumana et al. 2014). Glyphosate increases the amount of urea and uric acid in blood while causing the oxidative stress on the body which can lead to renal failure by damaging the renal tubular cells (VanDervort et al. 2014). Presence of glyphosate is reported in water of abandoned wells and surface water reservoirs in Padaviya, Sri Lanka where CKDu prevalence is high (Jayasumana et al. 2015a, b, c, d). High values of glyphosate concentration are reported from urine analysis of CKDu patients in Padavi-Sripura, Sri Lanka (Jayasumana et al. 2015a, b, c, d). 2,4-D (Hedanol) is another herbicide that has the ability to damage tubular cells effecting the renal function (VanDervort et al. 2014). Paraquat (a herbicide) can increase creatinine and uric acid level in blood by renal damage (VanDervort et al. 2014).

A number of fertilizers are used in agriculture such as urea, potash and phosphate fertilizers. Contribution of phosphate fertilizers in CKDu seems to got attention of many (Bandara et al. 2011; Dharma-wardana, 2018; Jayasumana et al. 2015c; Wanigasuriya et al. 2011; S. A. Wimalawansa & Wimalawansa, 2014). More research studies are in need exploring the contribution of other fertilizers as well. Phosphate fertilizers have many advantages such as enhancing the plant growth in agriculture. However, phosphate fertilizers can include heavy metals and arsenic. Chemical fertilizers such as TSP (triple super phosphate), a well-known phosphate fertilizer, are included with number of nephrotoxins such as As, Pb, Cd, Cr and Ni which can be chelated by glyphosate and dissolve into water easily (Jayasumana et al. 2015a, b, c, d). In El Salvador, nearly 16 million Kg of pesticides was imported during 2005–2010 resulting extremely high ratio of 2.5 kg of pesticides per person, while in 2010, 2,4-D 5.37 million Kg, Glyphosate 2.74 million Kg and Paraquet 0.81 million Kg were imported to El Salvador according to the Ministry of Economy database for imported pesticides (VanDervort et al. 2014). In 2012, Sri Lanka approved to import nearly 5.3 million Kg of glyphosate (Jayasumana et al. 2014).

### Heavy metals and excess trace elements

Nephrotoxic heavy metals (lead (Pb), cadmium (Cd), etc.) and arsenic (As) pose major threats to the human body. Usually, gastrointestinal tract, skin and inhalation are the main doors for entering heavy metals to human body. Heavy metals to the human body tend to bioaccumulate in the body. Heavy metals and arsenic interfere with the metabolic functions by damaging normal behaviour causing negative impact on the body. They can act as pseudoelements and disrupt cellular processes such as growth and damage-repairing (Balali-Mood et al. 2021; Jaishankar et al. 2014; Witkowska et al. 2021). Heavy metals especially target the kidney because the kidney can reabsorb and concentrate divalent ions and metals (Lentini et al. 2017). A study of urine analysis of CKDu patients and healthy controls in Padavi-Sripura (endemic area) and people in non-endemic areas in Sri Lanka was conducted for heavy metal exposure. The study evidences higher level of the multiple heavy metal exposure of the people in endemic region than in non-endemic and some of these heavy metals are Sb, As, Cd, Co, Pb, Mn, Ni, Ti and V (Jayasumana et al. 2015a, b, c, d). Phosphate fertilizers as well include heavy metals and arsenic (Jayasumana et al. 2015a, b, c, d). In Sri Lanka, some reports the presence of Pb and Cr in rice and Cd and Cr in water (Fernando et al. 2018; 2020). A limited study in 2012–2013 carried out in the North Central Province, Sri Lanka on the presence of heavy metals and trace elements biological samples (blood and hair) and environmental samples (rice, freshwater fish, soil and drinking water) revealed low-level exposure of Pb, Cd, and Hg of biological samples and exceeding concentrations of As, Cr, Cu, Fe, Hg, Mn, Ni, Pb and Se in soil as compared to mean background US criteria, while the concentrations of F, Fe, Mn, Na and Pb in drinking water samples exceeded the values recommended by the WHO. Furthermore, it reports a unique hydro geochemistry with high hardness and elevated fluoride content in CKDu areas (Levine et al. 2016). However, a study in Sri Lanka in 2016 reported no significant variation in toxic trace metals in hair and nail samples between CKDu cases and controls, ruling out the possibility of heavy metals being a causative factor for this disease (Diyabalanage et al. 2017). However, many reports show evidence of the involvement of heavy metals (mainly Cd and Pb), arsenic species, fluoride and hardness of the water in this disease. Even though many heavy metals in environmental samples show less values compared to WHO standards, they can be bioaccumulated in foods and human body posing a major threat (Cheryl Dybas, 2018; Noble et al. 2014).

### Usage of polluted water and polluted agricultural land

Due to the excessive use of agrochemicals, it has caused pollution of ground and surface water with negative impact on water resources and agricultural lands. Run off from agricultural lands contains lots of nephrotoxic agents which are mentioned above. Direct use of these polluted water for drinking, cooking and agriculture makes the way for bioaccumulation mainly in human body, animals (fish) and crops. The National Science Foundation (NSF) in Sri Lanka reports that it is suspected that drinking from polluted reservoirs and wells can be a major causal factor for CKDu. Further, it is reported that the use of alternative water sources helped in decreasing the CKDu rate (Cheryl Dybas, 2018).

Altitude also plays a suspected role in CKDu existence. A Costa Rican study reports that for every 200 m above sea level, CKDu prevalence increased by 26% (Harhay et al. 2016). In addition, usage of low-quality aluminium kitchen utensils, snake bites, cyanobacteria, genetic links and nephrotoxic mycotoxins are suspected to be causative factors for CKDu (Cheryl Dybas, 2018; Noble et al. 2014; Rajapakse et al. 2016).

### Mitigation steps for CKDu

No promising solutions are presented for CKDu situation so far. Mysterious nature and lack of early diagnosing techniques seem to be bottlenecks. However,  a number of research studies have been reported with some mitigation steps. Together with that, suggested mitigation steps are mentioned as follows. **Providing shelters and reducing strenuous labour**: Reducing the heat stress and cardiovascular demand can prevent the chronic dehydration decreasing the stress on kidneys. Following US OSHA guidelines for working under hot climate and conducting WRS (Water.Rest.Shade), intervention can be possible steps. (Heat—Standards | Occupational Safety and Health Administration, n.d., 2021; Heat Index—Introduction | Occupational Safety and Health Administration, n.d., 2021). Some studies among El Salvador sugarcane workers report the extreme occupational heat exposure and upon following WRS intervention, the symptoms of heat stress were decreased. If properly maintained, it is possible to conduct the intervention without reducing the work productivity. The researchers used Camelbacks for each worker to bring the water into fields and mobile canopy to provide shelters in the field (Bodin et al. 2016; Garcia-Trabanino et al. 2015). **Use of Organic fertilizers and Reduce the use of agrochemicals**: Synthetic or chemical fertilizers are the main source of most of the suspected nephrotoxins. Avoiding the use of low-quality agrochemicals and replacing those with environmentally friendly products will be a good approach to reduce the risk for CKDu. **Use of purified water:** This step has importance because the entire health delicately depends on the purity of water that is being consumed. One way of tackling the heat stress and chronic dehydration is the consumption of drinking water. However, the use of polluted drinking water can worsen the situation. Currently, the Sri Lankan Government has applied small-scale reverse osmosis plants for drinking water but in terms of maintenance and management due to membrane fouling, etc., this technology is unaffordable particularly for developing countries which are the most affected by CKDu (Cooray et al. 2019). Hence, addressing the hydrochemistry, the need to find cost-effective and eco-friendly novel treatments considering both, household purification and massive purification projects for agricultural water sources (rivers, lakes, etc.) are matters of concern. **Conducting awareness programmes and sufficient attention of world authorities**: This step also holds an important role because abundance of awareness and counselling programmes can interact with the affected communities in a friendly way and solve most of the psychological problems. Even though relevant authorities are not in action, being aware about workers’ rights and how to tackle the situation in an individual level (avoiding heat exposure, drinking sufficient amount of water, avoiding use of agrochemical and using standard procedures when dealing with the toxic chemicals to minimize environmental exposure, etc.) can make a major difference in communities effectively. Further, national and international level involvement is needed because mass production of some of the suspected causative factors can only be eliminated with international agreements since multi-national industrial companies are involved. Addressing this issue in the educational system since early age will motivate the active participation of the society in the solution of the problem. In addition, this is an excellent approach to encourage young people in pursuing careers in Science and Technology relevant to the needs of their affected countries**.** Within this context, the involvement of policymakers in all dissemination programmes is essential. It must be acknowledged that most countries in the developing world have policies and regulations regarding water purification and other environmental issues but the lack of technology unable their implementation. Therefore, collaboration at international level is required tending to reinforce academic-industrial links which are very limited in the developing world. More research studies for early detection methods, risk factors and circumventing steps need to be carried out exploring the related sectors such as anatomical pathology, geochemistry and environmental health. A number of organizations such as La Isla network are currently working on this, but more assistance of relevant world authorities can boost the works and contribute significantly to mitigate this disease. Figure 2 shows several suggested mitigation steps.

## Discussion

CKDu is an occupational endemic situation in most tropical countries, being a heavy burden to the world health economy. With the upcoming climate change, the situation can become more acute. This disease causes extremely painful experiences both mentally and physically. The CKDu is thriving among poor labouring and farming communities in tropical countries where their physical strength is the way of living. The disease involves progressive kidney damage offering a non- functional kidney. So, the patients will face fatigue, panting, lack of appetite, nausea, sleep disturbance, exhaustion, weight loss leading towards lack of physical strength. Due to lack of physical strength, it is not possible to continue their way of living. If the treatments are not in place, death is inevitable. But available treatments (RRT) are too much to afford. Fear of the death, being a burden to the family, and spending saved money for treatments, where hopes towards permanent cure is not reassured, make a significant psychological impact. More than 30,000 deaths are estimated per year. But the mysterious nature or lack of understanding of the root causes and early detection methods is the bottleneck for getting rid of this situation.

However, significant amount of research studies can be observed mainly in Mesoamerican and South Asian countries. CKDu is suspected as a multifactorial disease giving suspected causative factors such as heat stress, strenuous labour, dehydration, synthetic agrochemicals such as pesticides, herbicides and fertilizers, heavy metals and excess trace elements, the use of polluted water and polluted agricultural lands, low-quality aluminium kitchen utensils, snake bites, cyanobacteria, genetic links and nephrotoxic mycotoxins. More research studies need to be done to identify the exact causative factors. However, there is an utmost importance to act immediately to mitigate these causal factors because it can be seen that the disease is already causing a significant damage and some factors need years to get rid of. Above-mentioned causal factors can be divided into two main sections as water stress on the kidney and nephrotoxins. Heat stress, strenuous labour and dehydration cause the water stress on the kidney. Water plays a crucial role in removing waste products from the blood and in forming urine. Lack of water results in accumulation of waste products in blood leading to oxidative stress, kidney stones and urinary tract infections. Agrochemicals, heavy metals and arsenic can be included in the list of nephrotoxins. Heavymetals and arsenic can be added to the soil mainly through fertilizers such as TSP. In addition to being nephrotoxic by themselves, some agrochemicals such as glyphosate can act as chelating agents for nephrotoxic heavy metals and arsenic which are in soil and bring them into water. The latest suggestion in Sri lanka is the usage of hill country water which is polluted by agrochemicals used in hill country vegetations since Sri Lanka has a radial irrigation system starting from the middle hill country and flows towards the sea nourishing the lower lands. Further, flooding can cause the sorption of nephrotoxins to the soil. Rice cultivation that used either these lands or water will bioaccumulate arsenic and the heavy metals (mainly arsenic, cadmium and lead) in rice grains causing CKDu upon consumption (Diyabalanage et al. 2016). Central America also has a similar geography to Sri Lanka which includes both mountainous and coastal lands where the coastal region shows a higher CKDu prevalence. However, no reports can be found regarding similar situation as in Sri Lanka on hill country water usage.

By analysing the reported studies, it is clear that the polluted water plays an important role in CKDu. In order to get rid of the water stress on the kidney, one needs to intake more water. Since water sources are polluted, more water means more nephrotoxins. Since foods also contain bioaccumulated nephrotoxins, humans’ energy source too is not helping to solve the problem. Due to the global trade system, these foods or food products can be traded to other countries. Even if these products contain only a trace amount of heavy metals, consumption over long period of time will bioaccumulate in the human body causing severe health complications. Further, estimated climate change shows red signals on upcoming global temperature rise and water stress which make the situation more tragic. Available water sources as well are getting polluted rapidly due to anthropogenic activities. So, the tropically limited CKDu will engulf other countries without any delay.

The main point that can be observed is the silent nature of this killer. It was in the 1960s this first started to appear but it has taken many more decades to understand its true nature. Two main facts can be identified towards tackling CKDu. One is to conduct more research studies towards CKDu prevalence, detection and surveillance methods and exact root causes. For this, proper attention of the relevant world authorities and sufficient funding towards research studies are needed. This will be an investment towards world health economy in a way as well. Other fact is to deal with the current suspected causative factors. Some of suggested mitigation steps can be providing shelters and reducing strenuous labour, the use of organic fertilizers and reduce the use of synthetic agrochemicals, the use of purified water and conducting awareness programmes. However, the polluted soil and water will need years to be recovered even though synthetic agrochemicals get banned completely. Therefore, the proper water treatment is essential to get rid of relevant nephrotoxins. Unfortunately, only the reverse osmosis (RO) is able to deal with heavy metals among commercially applicable water purifying techniques currently available. Therefore, more research studies need to be put in place for cost-effective and eco-friendly water treatment methods. Considering all these facts, a multidisciplinary approach is required involving many parties such as nephrologists, environmentalists, geologists, chemists, engineers, politicians, and industrial owners working together towards eradication of the disease. Related biochemical and geochemical reactions should be well investigated. Above-mentioned relevant mitigation steps need to be in action immediately and more research studies are needed to be carried out finding early detection and surveillance methods, exact causative factors and mitigation steps.

## Conclusion

Chronic kidney disease of uncertain aetiology (CKDu) is a worldwide hidden health threat. It involves loss of kidney function progressively over a long period of time where absence of known risk factors of the normal chronic kidney disease (CKD) such as diabetes and hypertension while tubular proteinuria, absence of hypertension and oedema, characterize the disease clinically. Symptoms involve fatigue, panting, lack of appetite, nausea, sleep disturbance, exhaustion, weight loss and death. When diagnosed, renal replacement therapies (RRT: dialysis and kidney transplantation) are the only treatments available. Over last three decades, the disease has expanded to around 35 countries showing its rapid growth. CKDu is considered as a multifactorial disease giving suspected causative factors such as heat stress, strenuous labour, dehydration, exposure to synthetic agrochemicals (such as pesticides, herbicides and fertilizers), heavy metals and excess trace elements, and the use of polluted water and agricultural lands. However, exact roots of the disease are still uncertain. Suggested mitigation steps include (i) the provision of shelters (ii) reduction strenuous labour (iii) the use of eco-friendly agrochemicals (iv) the use of purified water. These steps should be accompanied by implementation of awareness programmes and granting sufficient attention of world authorities.

## Future prospects

More case studies should be done exploring more medical facts, worldwide prevalence, etc., to unfold the mysterious nature of the disease. Furthermore, more collaborative work needs to be implemented towards discovering early detection and surveillance methods, causative factors and mitigation steps of this deadly silent killer. Based on the information found so far, mitigation steps and awareness programmes should be put in place without any delay to halt the further growth of the disease.
